# Utility of diffusion-weighted imaging in differentiating benign and malignant breast lesions

**DOI:** 10.4102/sajr.v28i1.2952

**Published:** 2024-10-09

**Authors:** Allen Johnson, Radha Sarawagi, Rajesh Malik, Jitendra Sharma, Abhinav Bhagat

**Affiliations:** 1 Department of Neuroimaging and Interventional Radiology, National Institute of Mental Health and Neurosciences (NIMHANS), Bangalore, India; 2 Department of Radiodiagnosis and Imaging, All India Institute of Medical Sciences, Bhopal, India

**Keywords:** breast cancer, MRI, ADC, benign, malignant, BI-RADS, radiology

## Abstract

**Background:**

Breast cancer presents a significant global health burden. An accurate differentiation between benign and malignant lesions is imperative for timely intervention. While dynamic contrast enhanced MRI (DCE-MRI) is highly sensitive, its specificity is limited. This has led to the exploration of diffusion-weighted imaging (DWI) in distinguishing between benign and malignant breast lesions.

**Objectives:**

The study aimed to explore the diagnostic utility of DWI in distinguishing between benign and malignant breast lesions.

**Method:**

Assessment of 38 breast lesions using DWI with a *b* value of 800 s/mm^2^, performed with 3 Tesla MRI. The diagnostic performance of two different region of Interest (ROI) placement approaches was compared to obtain a feasible cut-off value of apparent diffusion coefficient (ADC) to differentiate between malignant and benign lesions. The histopathological reports were used as the gold standard.

**Results:**

ADC values of malignant lesions were significantly lower than those of benign lesions (0.84 × 10^−3^ mm^2^/s vs. 1.54 × 10^−3^ mm^2^/s). The average ADC measured using a small-sized 2D ROI including the darkest part in the ADC map, performed better than the large 2D ROI covering the entire lesion.

**Conclusion:**

Using a cut-off value of 0.98 × 10^−3^ mm^2^/s, ADC obtained high sensitivity (90%) and specificity (88.9%) in distinguishing between benign and malignant breast lesions.

**Contribution:**

Utilising quantitative analysis of DWI with ADC value measurement, reliably distinguished between benign and malignant breast lesions in this cohort, especially when employing a higher *b* value of 800 s/mm^2^.

## Introduction

Breast cancer is a major global health concern, causing substantial morbidity and mortality. Breast cancer now accounts for 1 in 8 cancer diagnoses worldwide and 2.3 million new cases in both sexes combined. It has surpassed lung cancer as the most prevalent disease diagnosed worldwide.^1^ The majority of the breast lesions detected are benign. Distinguishing between benign and malignant lesions is vital for precise and timely medical care. Early identification of malignancies allows swift intervention and treatment, reducing patient anxiety and avoiding unnecessary invasive procedures for those with benign conditions.^2^

MRI of the breast is a highly sensitive tool in breast cancer detection. MRI provides detailed breast tissue images, particularly valuable for assessing high-risk individuals, determining disease extent, and detecting tumours missed by mammography or ultrasound. Dynamic contrast enhanced MRI (DCE-MRI) has an 88% – 100% sensitivity.^3^ However, it has a lower specificity in assessing breast tumours, with difficulties in distinguishing benign lesions resembling malignancies and often resulting in unnecessary biopsies.^4,5^ These limitations impede widespread clinical utilisation and acceptance of MR imaging in various institutions.

Consequently, over the past decade, diffusion-weighted imaging (DWI) has become an imaging tool of interest for improving breast cancer detection and characterisation because of the complementary information about the microscopic cellular environment, ease in implementation of the technique, faster imaging time, availability on most commercial MRI scanners, and lack of the need for contrast media.

Literature reviews demonstrate DWI’s potential in distinguishing between benign and malignant breast lesions (Table 1). Nevertheless, current challenges lie in considerable variability in results, including diagnostic performance and apparent diffusion coefficient (ADC) cut-off levels. Additionally, inconsistent image quality related to diverse MRI system setups, sequences, and protocols globally impacts DWI’s perceived usefulness in breast MRI. Standardised acquisition protocols and interpretation guidelines are required to facilitate the clinical application of DWI and enable cross-institutional comparisons. However, it is noteworthy that there is a lack of widely accepted standard guidelines in this regard.

**TABLE 1:** Previous DWI breast imaging studies.

Studies	Year	MRI system	*b* value (s/mm^2^)	ADC value – benign (×10^−3^ mm^2^/s)			ADC value – malignant (×10^−3^ mm^2^/s)			ADC cut-off value (×10^−3^ mm^2^/s)
				Mean	s.d.	Median	Mean	s.d.	Median	
Kul et al.^6^	2014	Siemens 1.5 T	50, 400, 1000	1.21	0.36	-	0.75	0.19	-	0.90
Bansal et al.^7^	2015	GE 3T	0, 1000, 1500	1.35	-	-	0.89†	-	-	1.10
Ramírez-Galván et al.^8^	2015	GE 1.5T	700	1.41	0.22	-	0.87	0.12	-	1.08
Caivano et al.^9^	2015	Philips 3T	0, 750	2.06	0.19	-	1.03	0.07	-	NA
Wan et al.^10^	2016	Philips 1.5T	0, 1000	1.27	0.42	-	0.89	0.29	-	1.088
Yeong Yi An et al.^11^	2017	Siemens 3.0T	0, 750	1.14	0.23	-	0.88	0.19	-	1.0
Ebru Yılmaz et al.^12^	2018	GE 1.5T	0, 1000	1.584	-	-	0.884	-	-	1.04
Rahbar et al.^13^	2019	1.5T/3T	0, 100, 600, 800	1.47	0.29	-	1.21	0.21	-	1.53
Sehnaz Tezcan et al.^14^	2020	Siemens 1.5T	0, 800	-	-	1.03	-	-	0.72	0.89
Muzhen He et al.^15^	2021	Siemens 3.0T	0, 1000	1.26	0.21	-	0.80	0.09	-	0.983

*Source:* Please see full reference list of Johnson A, Sarawagi R, Malik R, Sharma J, Bhagat A. Utility of diffusion-weighted imaging in differentiating benign and malignant breast lesions. S Afr J Rad. 2024;28(1), a2952. https://doi.org/10.4102/sajr.v28i1.2952

ADC, apparent diffusion coefficient; DWI, diffusion-weighted imaging; GE, General Electric Heathcare; s.d., standard deviation; T, Tesla; NA, not applicable.

† , Mucinous carcinoma: 1.9.

The aim of this study was to distinguish between benign and malignant breast MRI lesions using DWI, and compare the diagnostic performance of varying region of interest (ROI) types to obtain a feasible ADC cut-off value to differentiate between malignant and benign lesions.

## Research methods and design

### Study design, setting and population

A prospective cross-sectional study was conducted at a tertiary care hospital in central India. Females older than 18 years of age, who had a mass lesion in the breast on MR imaging and subsequently had histopathological confirmation, were included. Individuals with prior breast surgery or chemotherapy, breast implant recipients, pregnant women, focus < 5 mm, and lack of histopathological diagnosis were excluded.

By considering previous similar studies and assuming mean ADC values of benign and malignant lesions to be (1.74 ± 0.46) × 10^−3^ mm/s^2^ and (1.25 ± 0.29) × 10^−3^ mm/s^2^, respectively, for a desired 95% confidence interval and 80% power, the required sample size calculated using the formula for sample size calculation for mean difference is 10 for each group (benign and malignant), anticipating almost equal number of benign and malignant breast lesions to be included in the study.^9^ Therefore, the required total sample size was 20 lesions. However, because of logistical reasons and anticipated loss to follow-up to review the histopathological diagnosis, we included all adult female patients who underwent breast MRI during a 1-year study period and met our inclusion criteria. A total of 26 women with 38 lesions met our inclusion criteria.

### MRI acquisition and interpretation

Utilising a dedicated 32-channel phased-array bilateral breast coil, we conducted the MRI on a 3T MRI system (Discovery MR750w, GE Healthcare). Patients were positioned in a prone posture with arms elevated above the head. For premenopausal patients, MRI examinations were conducted during the second week of their menstrual cycle. Each MR examination encompassed a T2-weighted sequence, a T1-weighted 3D non-fat-suppressed sequence, a T1-weighted DCE-MRI sequence, and a DWI sequence. All scans were executed in the axial orientation.

Axial DWI MR images were obtained before administering a gadolinium-based contrast material, utilising an echo-planar imaging (EPI) sequence. Parallel imaging with sensitivity encoding (ASSET) was employed with an acceleration factor of two. Additional parameters included fat suppression (Chemical Fat-Sat), volume shimming, *b* values of 0 and 800 s/mm^2^, 5690/73.8 (repetition time msec/echo time msec), a 250 Hz/pixel bandwidth, 4-mm section thickness, a 35 cm × 35 cm field of view, and a 192 × 192 matrix.

We used intravenous administration of Gadodiamide (Omniscan; GE Healthcare) as the contrast agent for DCE-MRI. The contrast was introduced as a 0.1 mmol/kg bolus using a power injector (Spectris Solaris; Medrad) at a rate of 3 mL per second, followed by a 10 mL saline flush. DCE-MRI captured sequential axial fat-suppressed high-resolution T1-weighted 3D fast gradient-echo images of both breasts (VIBRANT™). These images were acquired before contrast medium administration and at intervals of 68, 136, 203, 271, and 340 s later. The acquisition parameters were 4.8/1.7, 10° flip angle, 2-mm section thickness, 35 cm × 35 cm field of view, and 256 × 254 matrix.

The MRI scans of the breasts were reviewed and assessed for the presence of mass lesions and classification of the lesion’s morphology, encompassing shape and contours, its signal intensities on T1 and T2 images, and the pattern of intravenous contrast enhancement. Moreover, kinetic analysis on the mass was performed on the DCE-MR images to determine its size, defined as the largest diameter of the lesion.

Qualitative and quantitative assessments were conducted on the DWI and ADC images. Greyscale DWI and ADC maps were used to classify lesions exhibiting restricted diffusion for the qualitative evaluation. These were identified by observing high signal intensity on the DWI *b* = 800 image and signal loss on the corresponding ADC map.

**FIGURE 1:**
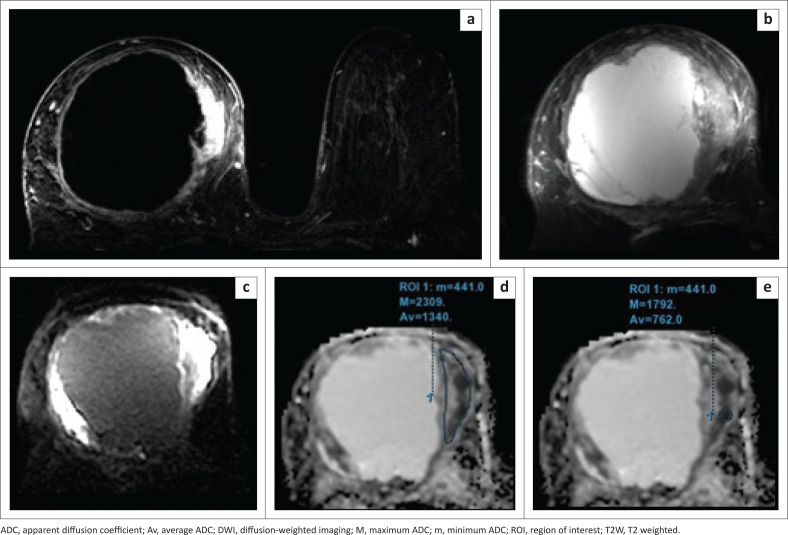
The different region of interest (ROI) placement techniques. (a, b) Subtraction and T2W images show a large solid-cystic lesion in the right breast with a predominant cystic component and eccentric enhancing solid component on the medial aspect of the lesion. (c) The solid component appears hyperintense on DWI (*b* = 800). (d) Large 2D freehand ROI drawn covering the solid component, avoiding the cystic or necrotic component of the lesion, which reveals minimum ADC = 0.44 × 10^−3^ mm^2^/s, maximum ADC = 2.3 × 10^−3^ mm^2^/s, and average ADC = 1.34 × 10^−3^ mm^2^/s. (e) Small 2D ROI drawn within the solid component covering only the part of the lesion appearing darkest on the ADC map reveals minimum ADC = 0.44 × 10^−3^ mm^2^/s, maximum ADC = 1.79 × 10^−3^ mm^2^/s, and average ADC = 0.76 × 10^−3^ mm^2^/s. Histology revealed infiltrating ductal carcinoma, Grade 3.

The average, minimum and maximum ADC values were calculated for quantitative evaluation by selecting the ROI within the lesion, avoiding areas affected by artefacts, necrosis, haemorrhage, non-enhancement and cystic degeneration. Two approaches were employed for placing the ROI (Figure 1), (1) a single freehand 2D-ROI was manually delineated, encompassing the entire lesion on the slice determined to exhibit the lowest ADC values (large 2D-ROI); and (2) a small 2D-ROI was manually drawn and positioned within the most constrained region within the solid portion on the ADC map (small 2D-ROI).

### Histopathological correlation

All of the patients were followed up for their histopathological outcomes. Histopathology specimens were obtained either by image-guided biopsy or surgery.

### Data analysis

Data were captured into Microsoft Excel version 16.51. Following data cleaning and rechecking, the data were transferred to Epi Info version 7.2 for statistical analysis. Data cleaning and rechecking involved checking for missing data, validating outlier ADC values to distinguish between data entry errors and valid measurements, ensuring consistency in ADC unit representation, and verifying adherence to the inclusion and exclusion criteria. For receiver-operating characteristic (ROC) curve analysis, online ROC software StAR was employed.^16^

Absolute frequencies and percentages were used for nominal data, like lesion type. Metric data, such as ADC, were presented using medians and interquartile ranges. Categorical variables, like menopausal status and lesion shape, were compared between benign and malignant groups through Chi-square or Fisher’s exact tests. The differences in ADC values between benign and malignant lesions were assessed using the Mann–Whitney *U* test. A significance level of *p* < 0.05 indicated statistical significance. The histological result was considered as the reference to assess DWI’s diagnostic accuracy. Receiver-operating characteristic analysis was used to compare the diagnostic performance of different ROI types and ADC parameters. This analysis helped determine a viable ADC cut-off value for distinguishing between malignant and benign lesions.

### Ethical consideration

An application for full ethical approval was made to the Institutional Human Ethics Committee, and ethics consent was received on 09 October 2019. The ethics approval number is IHEC-LOP/2019/MD0095. Written informed consent was obtained from all individual participants, ensuring their voluntary participation and understanding of the study’s purpose and procedures. The collected data were anonymised to safeguard confidentiality.

## Results

### Clinical demographics

Twenty-six women who collectively presented with 38 lesions that met our inclusion criteria participated in the study. The mean age of these patients was 41.3 years, with a standard deviation of 11.6 years and a range of 15–67 years. The histopathological diagnoses for 20 (52.6%) of the 38 lesions were malignant, which included 15 infiltrating ductal carcinomas (IDC), 1 mucinous carcinoma, 1 IDC with mucinous component, 1 infiltrating carcinoma with medullary features, 1 ductal carcinoma in situ (DCIS) arising within a phyllodes tumour and 1 malignant phyllodes tumour. The remaining 18 (47.4%) lesions were benign, which included 9 fibroadenomas, 1 giant fibroadenoma, 1 benign phyllodes tumour, 4 fibrocystic changes, 1 fibro-collagenous tissue, 1 usual duct hyperplasia and 1 granulomatous mastitis. The mean age of women with malignant lesions was significantly higher than those with benign lesions (Table 2).

**TABLE 2:** Patient characteristics.

Characteristic	Benign lesion group (*N* = 18)		Malignant lesion group (*N* = 20)		*p*

	Mean ± s.d.	*n*	Mean ± s.d.	*n*	
Patient age (years)	31.4 ± 11.7	-	45.1 ± 9.5	-	< 0.001*
Premenopausal women	-	15	-	9	0.014**
Postmenopausal women	-	3	-	11	0.014**

* , *p*-values were calculated by using *t* test.

** , *p*-values were calculated using *χ*^2^ test.

### Conventional MRI parameters

Significant distinctions emerged between benign and malignant lesions regarding lesion size, shape, margins, enhancement patterns and kinetic curves (Table 3). Most malignant lesions exhibited washout kinetics on DCE-MRI, with only one benign lesion (giant fibroadenoma) displaying such kinetics. The malignant lesions demonstrating persistent enhancement included mucinous carcinoma, DCIS originating within a phyllodes tumour and malignant phyllodes tumour.

**TABLE 3:** Conventional MRI lesion characteristics.

Lesion characteristic	Benign lesion group (*N* = 18)			Malignant lesion group (*N* = 20)			*p*
	Mean ± s.d.	*n*	%	Mean ± s.d.	*n*	%	
Lesion size (mm)	25.4 ± 28.9	-	-	53.1 ± 34.2	-	-	0.012*
**Lesion shape**	-	-	-	-	-	-	< 0.001**
Round	-	1	5.5	-	0	0.0	-
Oval	-	14	77.8	-	0	0.0	-
Irregular	-	3	16.7	-	20	100.0	-
**Lesion margin**	-			-			< 0.001**
Circumscribed	-	15	83.3	-	0	0.0	-
Irregular	-	3	16.7	-	10	50.0	-
Spiculated	-	0	0.0	-	10	50.0	-
**Lesion enhancement**	-	-	-	-	-	-	< 0.001**
Homogenous	-	4	22.2	-	0	0.0	-
Heterogenous	-	3	16.7	-	18	90.0	-
Rim	-	4	22.2	-	2	10.0	-
Dark Internal Septations	-	7	38.9	-	0	0.0	-
**Kinetic curve type**	-	-	-	-	-	-	< 0.001**
Type 1 – persistent	-	10	55.5	-	3	15.0	-
Type 2 – plateau	-	7	38.9	-	3	15.0	-
Type 3 – washout	-	1	5.6	-	14	70.0	-

s.d., standard deviation.

* , *p*-values were calculated by using *t* test.

** , *p*-values were calculated using *χ*^2^ test/Fisher’s exact test.

### Diffusion-weighted imaging

#### Qualitative DWI analysis

Upon visual examination of the *b* = 800 s/mm^2^ DWI images and their corresponding ADC maps, the majority of malignant lesions (80%) exhibited hyperintensity on the DWI *b* = 800 image, accompanied by signal loss on the corresponding ADC map (Table 4).

**TABLE 4:** Lesion appearance on DWI *b* = 800 s/mm^2^ image and ADC map.

Variable	Benign lesion group (*N* = 18)		Malignant lesion group (*N* = 20)		Sensitivity		Specificity		*p*

	*n*	%	*n*	%	%	95% CI	%	95% CI	
Lesion appearance on *b*800 images and ADC maps	-	-	-	-	80.0	55.7–93.4	66.7	41.1–85.6	0.004*
Restricted diffusion	6	33.3	16	80.0	-	-	-	-	-
No restricted diffusion	12	66.7	4	20.0	-	-	-	-	-

ADC, apparent diffusion coefficient; DWI, diffusion-weighted imaging; CI, confidence interval.

* , *p*-value was calculated using *χ*^2^ test.

#### Quantitative DWI analysis

**FIGURE 2:**
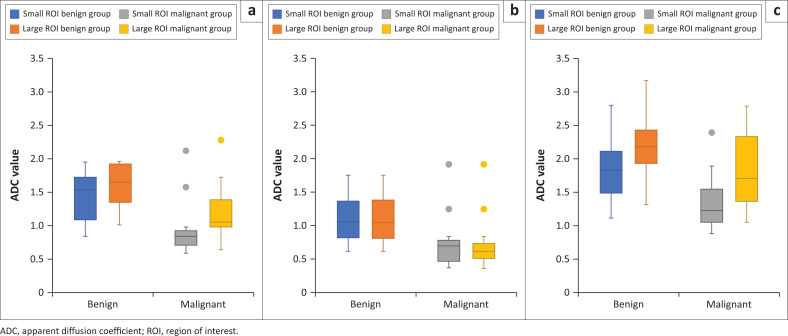
Boxplots showing (a) average, (b) minimum, and (c) maximum ADC values (×10^−3^ mm^2^/s) of small and large ROI placement approaches. ADC values of benign and malignant lesions were significantly different for both ROI approaches (*p* < 0.05).

The median values of the minimum, average and maximum ADC recorded for malignant lesions were significantly lower than those for benign lesions. This difference held for both the small and large 2D ROI placement approaches and was statistically significant (*p* < 0.05) (Table 5 and Figure 2).

**TABLE 5:** ADC values of benign versus malignant lesions by both region of interest methods.

ADC parameter (×10^−3^ mm^2^/s)	Benign lesion group (Median ± IQR)	Malignant lesion group (Median ± IQR)	*p*
**Small 2D ROI placement approach:**			
Average ADC	1.54 ± 0.55	0.84 ± 0.19	0.0001*
Minimum ADC	1.02 ± 0.51	0.59 ± 0.25	0.0014*
Maximum ADC	1.85 ± 0.54	1.1 ± 0.39	0.0005*
**Large 2D ROI placement approach:**			
Average ADC	1.64 ± 0.49	1.05 ± 0.39	0.0008*
Minimum ADC	1.02 ± 0.52	0.59 ± 0.20	0.0016*
Maximum ADC	2.19 ± 0.49	1.72 ± 0.84	0.0316*

ADC, apparent diffusion coefficient; IQR, interquartile range; ROI, region of interest.

* , *p*-values were calculated using the Mann–Whitney *U* test.

The small 2D-ROIs exhibited a 5.0 mm^2^–84.6 mm^2^ size range, with a mean value of 21.4 mm^2^ ± 15.9 mm^2^. Meanwhile, the large 2D-ROIs spanned from 18.6 mm^2^ to 7351 mm^2^, with a mean value of 592.5 mm^2^ ± 1507.9 mm^2^.

**FIGURE 3:**
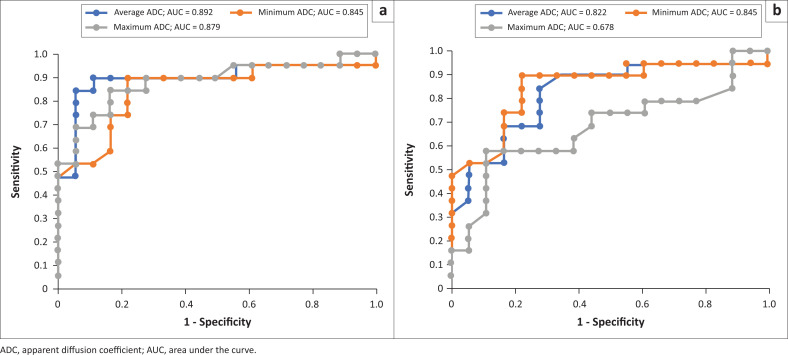
(a, b) Graphs show a comparison between receiver-operating characteristic curves for average, minimum and maximum ADC values obtained by both (a) small and (b) large region of interest approaches. Average ADC using small 2D region of interest has the highest area under the curve.

In terms of the areas under the ROC curve (AUC), the range extended from 0.678 (maximum ADC of large 2D ROI) to 0.892 (average ADC of small 2D ROI). Among the six ADC parameters, the average ADC using the small 2D ROI showcased the most robust performance, exhibiting the largest AUC (Figure 3). Conversely, the performance of maximum ADC using the large 2D ROI was significantly weaker.

The optimal ADC cut-off value (for average ADC using small 2D ROI) was determined to be 0.98 × 10^−3^ mm^2^/s, achieving a sensitivity of 90.0%, specificity of 88.9%, and an AUC of 0.89 (Table 6).

**TABLE 6:** Optimal ADC cut-off values from receiver-operating characteristic curves.

ADC parameter	Area under ROC curve	Cut-off level (×10^−3^ mm^2^/s)	Sensitivity (%)	Specificity (%)
**Small 2D ROI placement approach:**				
Average ADC	0.892	0.98	90.0	88.9
Minimum ADC	0.845	0.81	89.5	77.8
Maximum ADC	0.875	1.45	84.2	83.3
**Large 2D ROI placement approach:**				
Average ADC	0.822	1.42	84.2	72.2
Minimum ADC	0.845	0.81	89.5	77.8
Maximum ADC	0.678	1.75	57.9	88.9

ADC, apparent diffusion coefficient; ROI, region of interest; ROC, receiver-operating characteristic.

**FIGURE 4:**
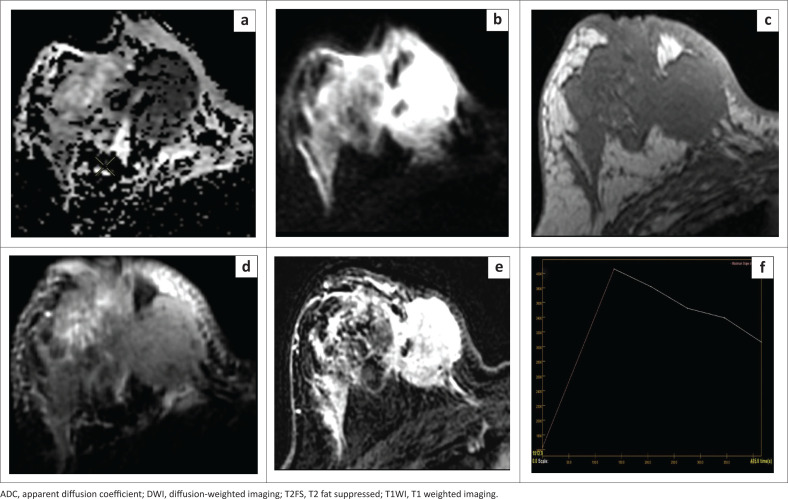
A 29-year-old female presented with a lump in her right breast. Histology revealed infiltrating ductal carcinomas, Grade 3, ER+, PR+, Her2–. (a) The lesion appears very hypointense on ADC map with a very low ADC value of 0.58 × 10^−3^ mm^2^/s. (b) The lesion appears hyperintense on DWI (*b* = 800). (c) Intermediate signal intensity on T1WI. (d) Intermediate signal intensity on T2FS. (e, f) Subtraction image shows heterogenous post-contrast enhancement with washout kinetics.

**FIGURE 5:**
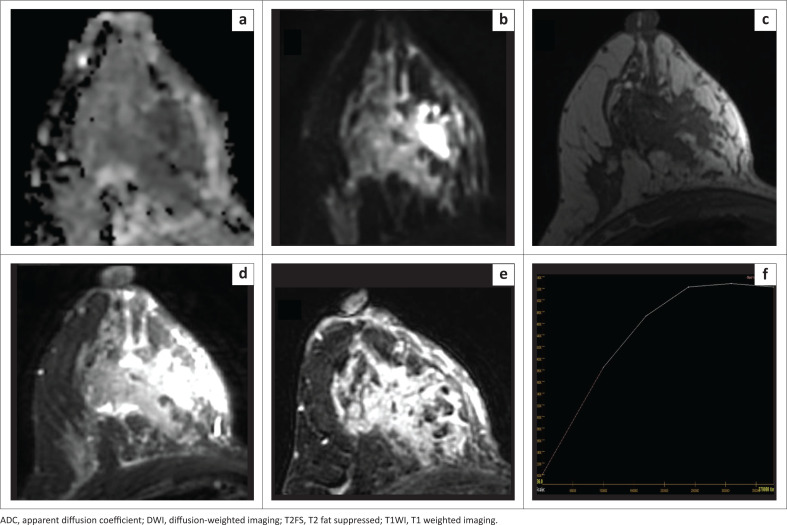
A 32-year-old female with a lump in the upper inner quadrant of her right breast with intermittent pain. Histology revealed granulomatous mastitis. (a) Patchy areas of reduced signal intensity on ADC map noted in the upper inner quadrant of the right breast with a low ADC value of 0.83 × 10^−3^ mm^2^/s. (b) Corresponding areas appear hyperintense on DWI (*b* = 800). (c) Intermediate signal intensity on T1WI. (d) hyperintense on T2FS. (e) Subtraction image shows heterogenous post-contrast enhancement. (f) Type II curve seen on kinetics analysis.

We found 20 lesions to be at or below this cut-off ADC value, of which 18 were malignant (true-positive), an example of which is depicted in Figure 4. However, two lesions were benign (false-positive), which included a case of granulomatous mastitis with an ADC value of 0.83 × 10^−3^ mm^2^/s (Figure 5), and a case of giant fibroadenoma with an ADC value of 0.96 × 10^−3^ mm^2^/s.

**FIGURE 6:**
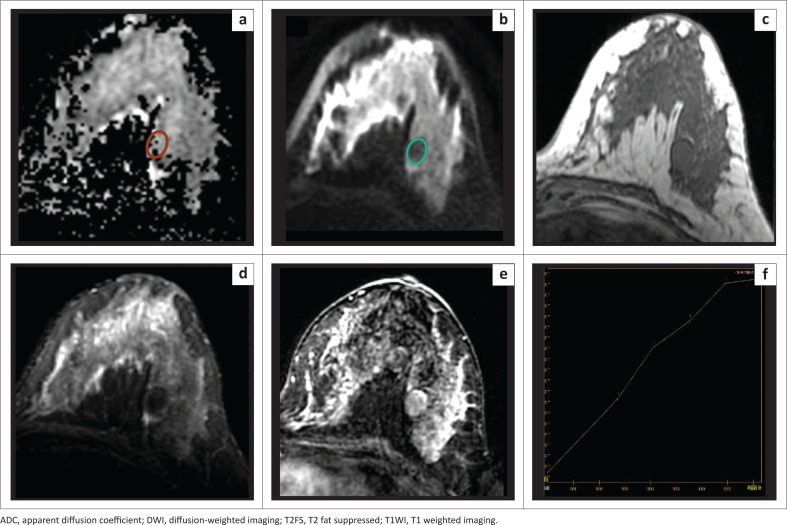
A 29-year-old female with a small palpable lump in her left breast. Histology revealed a fibroadenoma. (a) The lesion is not well visualised on ADC map. ADC value obtained by propagating the region of interest drawn on DWI is 1.68 × 10^−3^ mm^2^/s. (b) The lesion appears hypointense on DWI, *b* = 800 (Lesion depicted within the region of interest). (c) Intermediate signal intensity on T1WI. (d) Hypointense on T2FS. (e) Mild enhancement noted on the early subtraction image. (f) Persistent enhancement noted on kinetics analysis.

**FIGURE 7:**
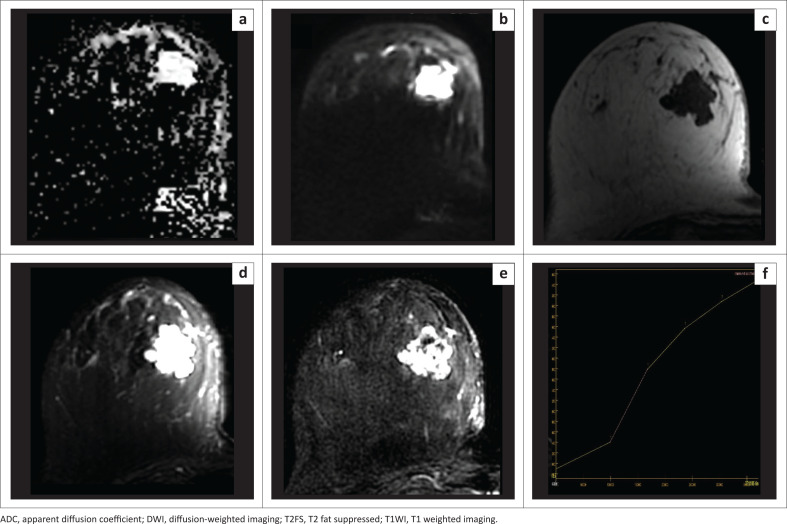
A 53-year-old female presented with a lump in the lower inner quadrant of her right breast. Histology revealed mucinous carcinoma. (a) The lesion appears hyperintense on the ADC map with a very high ADC value of 2.12 × 10^−3^ mm^2^/s. (b) The lesion appears hyperintense on DWI (*b* = 800). (c) Hypointense signal intensity on T1WI. (d) Hyperintense signal intensity on T2FS. (e, f) Subtraction image shows heterogenous post-contrast enhancement with persistent enhancement on kinetics analysis.

We found 18 lesions to have ADC values higher than the cut-off, of which 16 were benign (true-negative), an example of which is depicted in Figure 6. However, two were malignant (false negative), which included a case of mucinous carcinoma with an exceptionally high ADC value of 2.12 × 10^−3^ mm^2^/s (Figure 7). The other false negative was DCIS arising within a phyllodes tumour, displaying an ADC value of 1.57 × 10^−3^ mm^2^/s.

## Discussion

This study highlights the value of quantitative ADC analysis for distinguishing malignant and benign breast lesions. Qualitative assessment using DWI and ADC maps showed high sensitivity (80.0%) but lower specificity (66.7%), affecting its ability to differentiate between malignancies and benign cases. In contrast, quantitative analysis revealed a significant difference in ADC values between malignant and benign lesions (0.84 × 10^−3^ vs. 1.54 × 10^−3^ mm^2^/s). Using a cut-off value of 0.98 × 10^−3^ mm^2^/s ADC (average ADC using small 2D ROI), high sensitivity (90%) and specificity (88.9%) was achieved in accurately distinguishing between benign and malignant breast lesions.

Prior research consistently emphasises the significant contrast in ADC values between benign and malignant breast lesions. The diagnostic significance of low ADC values in detecting breast cancer suggests that these values correspond to the most malignant areas of the tumour. Although the current study did not establish a direct histopathological correlation, Guo et al. noted an intriguing observation. They found a consistent inverse relationship between ADC values and cellular density in breast lesions.^17^

The present study highlights how ROI placement influences ADC measurements in breast tumours. Small 2D ROIs for average ADC outperformed large 2D ROIs for maximum ADC in distinguishing benign from malignant lesions, reflecting the intrinsic structural variability in breast lesions impacting ADC values. Similar findings were observed in rectal cancer studies.^18^ Prior research consistently reveals significant contrasts in breast lesions’ ADC values between small and large 2D-ROIs. Smaller ROIs consistently perform better than larger ones.^19,20^

The choice of the ROI placement method significantly impacts DWI’s diagnostic performance for breast lesions and other tumours. Interestingly, maximum ADC, an often-overlooked parameter, exhibited notably poorer performance in this study when compared to minimum and average ADC. This trend aligns with results from a recent study by Hirano et al.^21^ This phenomenon can be attributed to the structural heterogeneity commonly found in breast lesions, resulting in the inclusion of less malignant or even necrotic lesion components. This inclusion can alter the maximum ADC measurements. Additionally, ADC measurements are susceptible to errors from noise generated by neighbouring voxels containing fat. Larger ROIs pose a higher risk of inadvertently measuring areas with suppressed fat, necessitating careful ROI placement.

The DWI protocol utilised in this study followed the European Society of Breast Imaging (EUSOBI) international breast DWI working group’s recommendation, utilising a high *b* value of 800 s/mm^2^. Notably, adherence to this recommendation is not common in existing studies. A prospective multicentre study (A6702) conducted by the ECOG-ACRIN Cancer Research Group in 2019 reinforces our findings. Their study found that malignancies had lower ADC values than benign lesions (1.21 × 10^−3^ vs. 1.47 × 10^−3^; *p* < 0.0001), and using an ADC cut-off of 1.53 × 10^−3^ mm^2^/s reduced biopsy rates by 20.9%.^13^ They reported relatively higher ADC values (0.84 × 10^−3^ vs. 1.54 × 10^−3^, cut-off value: 0.98 × 10^−3^), likely because of whole lesion ROIs. In contrast, our study followed the recommendation of utilising a small ROI placed in the darkest lesion region on the ADC map. This approach improves accuracy in distinguishing malignant and benign breast lesions, explaining the observed differences in ADC values.^22^

ADC values help distinguish benign and malignant lesions using an appropriate cut-off value. However, there can be an overlap in ADC values between these lesions, leading to potential false-positive and false-negative results. Using the ADC cut-off value established here, this study encountered two false-negative cases among the 20 malignant lesions assessed. One was mucinous carcinoma, which showed an unusually high ADC value of 2.12 × 10^−3^ mm^2^/s. This finding is consistent with a study by Woodhams et al., where the mean ADC of mucinous carcinoma (1.8 × 10^−3^ mm^2^/s) was statistically higher than that of benign lesions (1.3 × 10^−3^ mm^2^/s) and other malignant tumours (0.9 × 10^−3^ mm^2^/s) (*p* < 0.001). The low signal intensity of mucinous carcinoma on diffusion-weighted images likely reflects its mucin content and low cellularity.^23^ The other false-negative case involved DCIS arising within a benign phyllodes tumour. Conventionally, pure DCIS displays higher ADC values compared to invasive cancer. This observation aligns with the findings of Bickel et al., who highlighted a significant difference in ADC values between invasive cancers and non-invasive DCIS (0.9 × 10^−3^ vs. 1.24 × 10^−3^; *p* < 0.001).^24^

Fang et al.’s study revealed that the average ADC of phyllodes tumours was notably higher compared to fibroadenomas and malignant lesions. Malignant phyllodes tumours exhibited a higher ADC than their benign or borderline counterparts. This might be attributed to the fact that the ADC in malignant phyllodes tumours is influenced not only by tumour cell density but also by factors such as necrosis, cystic degeneration, and oedema within the tumour. Increased necrosis and oedema enable water protons to move more freely, significantly affecting the ADC value.^25^

In the evaluation of 18 benign lesions, we identified two false-positive outcomes. One entailed idiopathic granulomatous mastitis (IGM), characterised by restricted diffusion and a low ADC value of 0.83 × 10^−3^ mm^2^/s. These findings align with a retrospective study conducted by Fazzio et al., which showcased the fact that the affected parenchyma in IGM consistently exhibits restricted diffusion, marked by consistently lower mean ADC values (1.0 × 10^−3^ mm^2^/s) compared to normal breast parenchyma (2.3 × 10^−3^ mm^2^/s). This discrepancy might be because of the chronic inflammatory response in IGM, leading to diminished water diffusion capacity and reduced relative ADC values.^26^ The other false-positive case pertained to a giant fibroadenoma, presenting with an ADC value of 0.96 × 10^−3^ mm^2^/s. Notably, this lesion was sizable (largest dimension of 13.1 cm) and displayed heterogeneity, featuring multiple punctate foci characterised by low ADC values. While fibroadenomas are generally expected to display high ADC values because of stromal myxoid changes, Parsian et al.’s study involving 26 fibroadenomas revealed a distinct pattern. They observed that fibroadenomas with epithelial hyperplasia exhibited notably lower ADC values emphasising the impact of histopathological characteristics on ADC values in fibroadenomas.^27^ Consequently, the tiny punctate foci demonstrating restricted diffusion within the giant fibroadenoma might indicate regions of epithelial hyperplasia or potential carcinoma evolving within the fibroadenoma. It is plausible that the extensive size of the lesion led to the omission of these areas in histopathological analysis, underscoring the issue of under-sampling.

This study exhibits notable strengths, aligning with the recommendations of the EUSOBI^22^ by employing a high *b* value of 800 s/mm^2^, thereby contributing to international efforts for standardising breast DWI practices. The study was conducted on a 3T system with a 32-channel breast coil that provided better signal-to-noise ratio and spatial resolution with shorter imaging time. Moreover, the study’s recognition of the impact of ROI placement on ADC measurements in breast tumours is a significant strength. The findings emphasise that employing a small 2D ROI, capturing the darkest part in the ADC map, outperforms larger 2D ROIs, highlighting the internal structural heterogeneity of breast lesions. This nuanced understanding contributes valuable insights into the intricacies of ADC measurements, which are crucial for accurately differentiating between benign and malignant breast lesions.

One limitation of this study was the absence of patients with pure DCIS and lobular carcinomas, which typically manifest as non-mass enhancement on breast MRI. These entities can pose challenges in ADC measurement. Prior investigations have demonstrated that the ability of ADCs to differentiate between benign and malignant breast lesions is not as reliable for non-mass lesions compared to mass lesions.^6,28^ This study showed less overlap in ADC values between benign and malignant lesions. This could be because some specific malignant lesions were absent, like pure DCIS, which tend to have higher ADC values. It is worth mentioning that our study population did not come from a screening group. Additionally, our study had slightly more malignant cases than benign ones, primarily because our institution is a tertiary care centre.

## Conclusion

The current study demonstrates the efficacy of quantitative DWI analysis using ADC measurements in distinguishing between benign and malignant breast lesions on a 3T MRI system, particularly with a high *b* value of 800 s/mm^2^. This approach improves lesion characterisation accuracy, enhancing MRI’s specificity and diagnostic precision. Region of Interest placement significantly impacts ADC values, with average ADC from a small 2D ROI in the darkest ADC map region proving superior to large 2D ROIs.

We recommend further studies using standardised DWI protocols to refine our understanding, aiding in establishing an optimal ADC threshold.
